# Comparison of self-sampling blood collection for N-glycosylation analysis

**DOI:** 10.1186/s13104-022-05958-9

**Published:** 2022-02-16

**Authors:** Ana Cvetko, Marko Tijardović, Iva Bilandžija-Kuš, Olga Gornik

**Affiliations:** 1 Faculty of Pharmacy and Biochemistry, University of Zagreb, Ante Kovačića 1, 10000 Zagreb, Croatia; 2 University Hospital Centre Zagreb, 10000 Zagreb, Croatia

**Keywords:** Self-sampling, Glycan analysis, Plasma N-glycosylation, Comparability, Repeatability

## Abstract

**Objective:**

Self-sampling of capillary blood provides easier sample collection, handling, and shipping compared to more invasive blood sampling via venepuncture. Recently, other means of capillary blood collection were introduced to the market, such as Neoteryx sticks and Noviplex cards. We tested the comparability of these two self-sampling methods, alongside dried blood spots (DBS), with plasma acquired from venepunctured blood in N-glycoprofiling of total proteins. We have also tested the intra-day repeatability of the three mentioned self-sampling methods. Capillary blood collection with Neoteryx, Noviplex and DBS was done following the manufacturers’ instructions and N-glycoprofiling of released, fluorescently labelled N-glycans was performed with ultra-performance liquid chromatography.

**Results:**

Comparability with plasma was assessed by calculating the relative deviance, which was 0.674 for DBS, 0.092 for Neoteryx sticks, and 0.069 for Noviplex cards. In repeatability testing, similar results were obtained, with Noviplex cards and Neoteryx sticks performing substantially better than DBS (CVs = 4.831% and 7.098%, compared to 14.305%, respectively). Our preliminary study on the use of Neoteryx and Noviplex self-sampling devices in glycoanalysis demonstrates their satisfactory performance in both the comparability and repeatability testing, however, they should be further tested in larger collaborations and cohorts.

**Supplementary Information:**

The online version contains supplementary material available at 10.1186/s13104-022-05958-9.

## Introduction

In recent years, there has been an exponential growth in the development of precision-based diagnostics around the world. Additionally, the field of glycoscience [1] also experienced the tremendous growth and imposed its potential in the field of diagnostics and personalized medicine [2]. Since glycoanalysis and other specialized diagnostic testings are often performed in facilities inaccessible to either patient or their physician, there is a necessity of having a fast, reliable, and simple sample collection and handling prior to long-distance shipping. Glycoscience is also frequently associated with a high-throughput analysis of large number of samples from international studies. Most of these analyses are usually performed on a plasma or full blood, which requires invasive venepuncturing performed in a professional health care environment. Thus, translation to a more accessible sampling approach would be of great value.

Glycosylation, as one of the most common post-translational modification [3, 4], is involved in many physiological processes, as well as modification of both protein structure and function [5–8]. Many studies have investigated glycan changes in various disorders, such as inflammation, aging, cancer, autoimmune diseases [9–11]. Studies have reported glycans as very responsive to mentioned conditions, sometimes even years before its onset [12, 13], making glycans powerful potential biomarkers.

Plasma and whole blood differ due to the whole blood containing red and white blood cells and platelets, thus both cellular proteins as well as soluble plasma proteins [14]. However, studies on glycosylation and glycan structural annotation of both DBS and plasma display great similarities in structures and profiles obtained from whole blood and plasma, with majority of the structures matching in both samples [15–17].

A common standard for self-sampling of capillary blood are dried blood spots (DBS) [18]. Recent studies have characterized DBS as an adequate collection option for glycoprofiling [19, 20], however, lower intensities and poorer repeatability were observed [21, 22] compared to classic plasma samples. Since accessibility and non-invasiveness of DBS were welcomed [23] in the scientific and biomedical community, the field of self-sampling has started to grow rapidly. Neoteryx and Shimadzu have both presented their versions of self-sampling protocols for capillary blood. Neoteryx introduced Mitra sticks [24], which rely on their patent-protected volumetric sampling technology, VAMS, and absorb a constant volume of capillary blood (10 µL), while Shimadzu announced their Noviplex cards [25], used for acquiring dried plasma disks by membrane separation from capillary blood.

There are certain limitations of the self-sampling methods. One of the main shortcomings of the DBS is the variation of the spotted volume of capillary blood as well as the difference in the haematocrit (Hct) [26]. The difference in Hct on the surface of the blood spots poses a great challenge when puncturing the filter card to gather the DBS for analysis. To surpass this issue, Neoteryx Mitra sticks collect a constant blood volume, and the entire stick tip covered in whole blood is used for analysis [24]. Shimadzu Noviplex cards open great new possibilities to self-sampling with the option of separating plasma from capillary blood directly on the sampling card. Plasma separation resolves the problem of haematocrit, however, not all analytes can be analysed in plasma [26]. Also, Noviplex cards result in low amount of dried plasma (up to 3.8 µL) [25]. It is also important to mention that unwary sampling, such as too strong puncturing of the finger or excessive squeezing to extrude blood can result in haemolysis and interstitial fluid leakage which can influence the results of the analysis, while in Noviplex cards haemolysis can also decrease the efficacy of plasma separation.

In our study, we have compared glycoprofiles acquired by DBS, Neoteryx Mitra sticks and Noviplex plasma cards with our standard glycoprofile from venepuncturing-collected plasma sample to identify a method that would facilitate clinical implementation of the potential glycan biomarkers suggested by abovementioned studies. We have also tested in day repeatability of these self-sampling options.

## Main text

### Methods

#### Sampling and sample preparation

Blood samples were collected using four approaches: plasma was separated by centrifuging full blood collected in EDTA tubes after venepuncturing, while capillary blood was collected by Neoteryx Mitra sticks (Neoteryx, USA), DBS (Whatman, USA) and Noviplex cards (Shimadzu, Japan). DBS were left to dry at room temperature, while for Noviplex cards, after 3 min, the top layer was peeled, and the remaining plasma-containing disks were left to dry at room temperature for 15 min. Sampling by Neoteryx Mitra sticks was also done following the manufacturer protocol. After their entire tip was covered with blood (10 μL), sticks were left at room temperature to dry. After several hours, all samples were sealed in plastic bags and stored at 4 °C overnight. Glycoprofiling of all samples was performed the next day. For the glycoprofiling from plasma, 10 μL of plasma was used. Plasma samples were transferred to their designated positions on 96-well plates, assessed by manual randomization. Plasma disks, from the Noviplex cards, were also transferred to their positions, and 10 μL of ultra-pure water was added to each well containing the disk. DBS were first cut into circles approximately 6 mm in diameter, and then transferred to their positions on a plate, where they were also covered with 10 μL of ultra-pure water. Blood from Neoteryx Mitra sticks was firstly extracted following the manufacturer’s recommendations by putting the stick in 20 μL of ultra-pure water and shaking for 45 min at room temperature. After that, 10 μL of extracted solution for each sample was transferred to their position on a plate.

##### Study participants

Participants are employees of the Faculty of Pharmacy and Biochemistry (both medical and non-medical background, with majority of the participants being familiar with self-sampling procedures) where the study was conducted and performed. The participants were recruited after planning the experiment and their blood was sampled immediately prior to the execution of the experiment. Sample size was determined by participants’ availability for sampling throughout the entire study duration, as well as material availability. Self-sampling of each participant was performed for DBS, Mitra (Neoteryx) and Noviplex cards, while plasma gathered by venepuncturing was done by a healthcare professional. Total number of sampled participants is 10 (3 females, 7 males). Participants’ age range is 25–45 years of age, and all participants are considered healthy, absent of any medical diagnosis.

##### Comparability testing

We collected blood from all 10 individuals by four abovementioned approaches for the comparability testing. The protocol of sample acquiring, extraction and preparation was done as explained in the Sampling section. The plate also contained two blank samples and four standard plasma samples to monitor the quality of sample preparation procedure.

##### Repeatability testing

We collected blood from 5 out of 10 individuals (1 female, 4 males), for the repeatability testing, by puncturing ring and/or middle finger to acquire capillary blood for Neoteryx Mitra sticks, DBS and Noviplex cards. All individuals gave blood in hexaplicates for each self-sampling method. The protocol of sample acquiring, extraction and preparation was done as explained in the Sampling section. The plate also contained one blank sample and five standard plasma samples to monitor the quality of sample preparation procedure.

#### Deglycosylation, labelling and clean-up of plasma N-glycans

The entire sample preparation protocol for HILIC-UPLC-FLR analysis of labelled plasma N-glycans was done as described previously [27] and in the Additional file 1. This protocol was done in the same way for plasma samples, plasma disks obtained from Noviplex cards, DBS and blood extracted from Neoteryx Mitra sticks. Noviplex plasma disks and DBS remained in the wells until the SPE clean-up step.

#### HILIC-UPLC-FLR plasma N-glycan analysis

Labelled N-glycans separation was done by hydrophilic interaction liquid chromatography (HILIC) on Acquity UPLC H-Class instrument (Waters, USA) as explained previously [27] and in the Additional file 1. Although several small chromatographic peaks were noticed at the beginning of the whole blood chromatograms compared to plasma chromatograms, we integrated only the matching part, thus N-glycan structures originating from plasma proteins, in order to assure the direct comparison. We started with the core fucosylated bianntenary structure previously reported in both plasma and the whole blood N-glycome analysis [15–17]. Chromatograms were separated into 39 glycan peaks (GP). Previous publication has assigned structures in plasma protein N-glycosylation [16, 17], and the same structures were noticed in publication characterizing glycan structures in whole blood [15]. Representative chromatograms are available in the Additional file 1: Fig. S1–4. Glycan structures corresponding to peaks with detailed description is also available in the Additional file 1.

#### Statistical analysis

All statistical analysis and visualization of the results were performed in Microsoft Excel software (Microsoft, USA) as described in Additional file 1.

### Results

#### Comparability with plasma

**Fig. 1:**
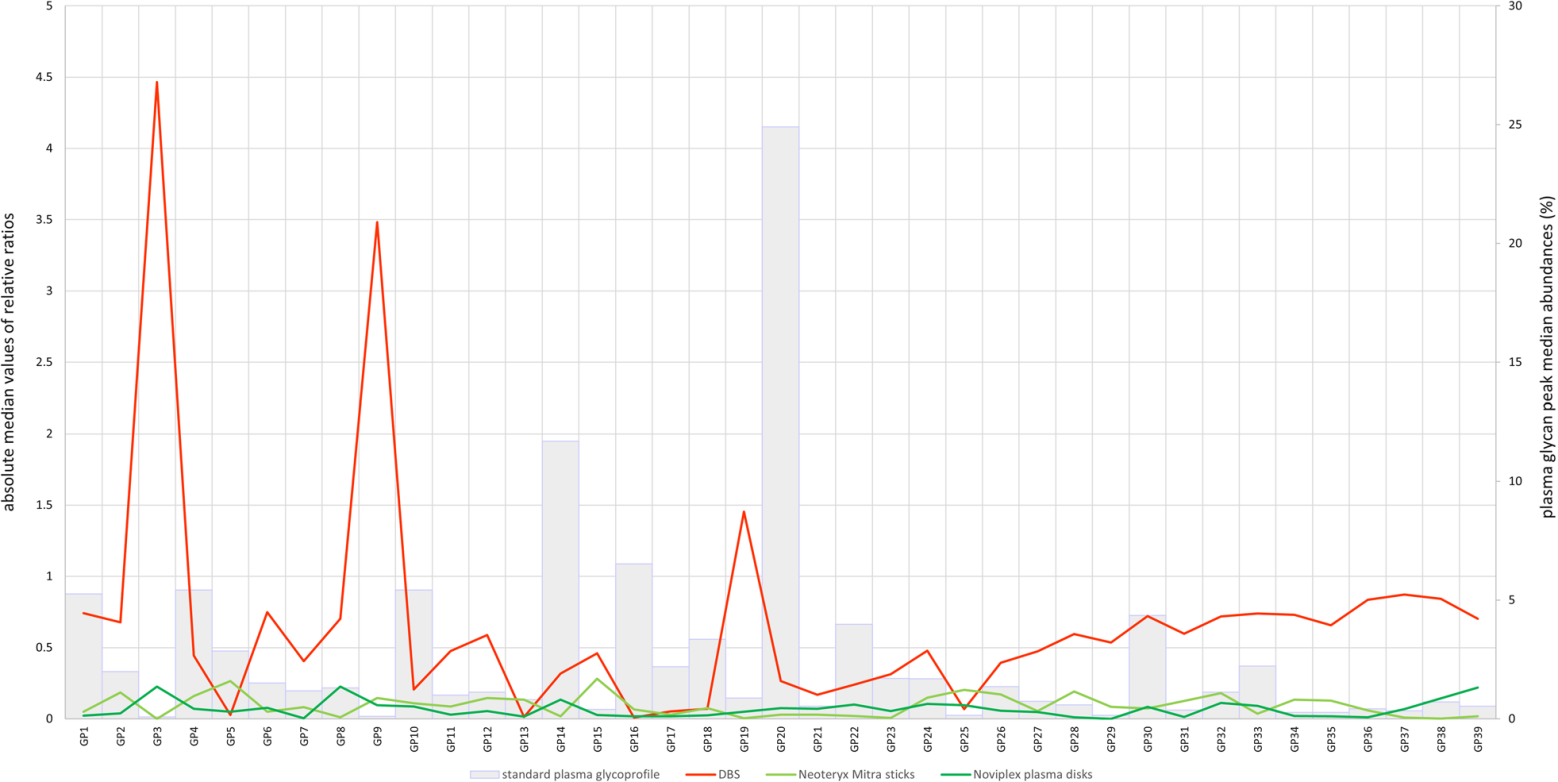
Comparability of self-sampling methods with standard plasma method. Median relative abundances of plasma glycan peaks are presented with grey bars. Absolute median values of relative ratios are presented with lineplots

In order to test if our glycan analysis approach can be complemented with self-sampling methods, we have decided to compare the results with our standard procedure, glycoprofiling of plasma proteins after standard plasma sampling from venepuncturing. To test the comparability of DBS, Neoteryx Mitra sticks and Noviplex plasma disks we have calculated relative deviance between the tested method and plasma (standard method) for each of the 39 glycan peaks (Fig. 1, Additional file 1: Table S2). The average relative deviance for DBS was 0.674, while for Neoteryx Mitra sticks is 0.092, and for Noviplex plasma disks is 0.069.

#### Repeatability of each method

**Fig. 2:**
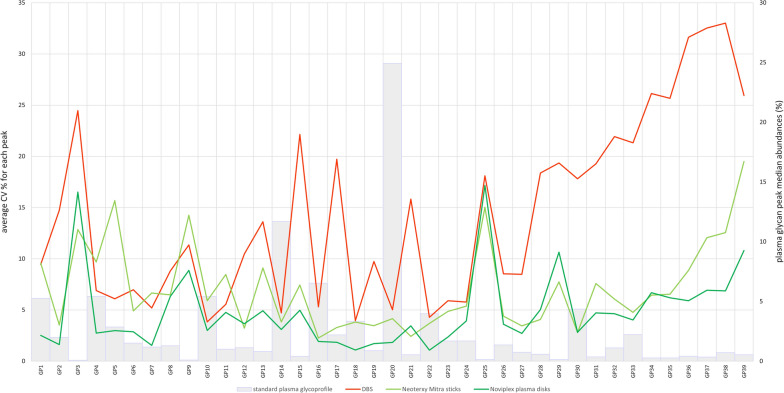
Comparison of repeatability between dried blood spots (DBS), Neoteryx Mitra sticks, and Noviplex plasma disks presented with lineplots of CV values (%). Median relative abundances of plasma glycan peaks are presented with grey bars

After testing the comparability of presented self-sampling methods with the standard plasma sampling, we wanted to check the repeatability of the glycan results obtained after each self-sampling procedure. Median CV % for each of the 39 glycan peaks calculated from hexaplicates from 5 participants are shown in Fig. 2. The average CV% of all peaks for DBS was 14.305%, for Neoteryx Mitra sticks 7.098%, and for Noviplex plasma disks 4.831%.

### Discussion

To test the comparability of self-sampling protocols with glycan analysis from venepuncture-gathered plasma samples, as well as their repeatability, we have chromatographically profiled 70 samples of released, fluorescently labelled N-glycans.

Comparability of the tested self-sampling protocols was assessed by calculating the average relative deviance from the standard procedure (plasma from venepuncturing). Noviplex plasma disks and Neoteryx Mitra sticks both performed well with their relative deviances averaging at 0.069 and 0.092, respectively. DBS showed the poorest comparability with an average relative deviance of 0.674. As expected, this comparability was less good for the smallest peaks whose relative abundance was less than 1%. However, both Neoteryx Mitra and Noviplex plasma disks managed to adequately replicate the plasma glycoprofile even for these small peaks.

Method repeatability for the self-sampling protocols was assessed by calculating the CV % for all glycan peaks (Fig. 2.). Again, Noviplex plasma disks and Neoteryx Mitra sticks performed well with average CV of 4.831% and 7.098%, while DBS had a lower repeatability of 14.305%.

Good performance of Noviplex plasma disks and Neoteryx Mitra sticks, both in comparability and repeatability testing, on a small-scale study like ours displays their great potential for inclusion in larger studies on bigger cohorts. Not only are these methods less invasive and easier for the patients, but they are also quicker and more accessible for healthcare professionals and scientists. Also, studies have shown high similarity between whole blood and plasma glycosylation profile. Therefore, comparison of glycosylation analysis using these methods is highly welcomed. On the other hand, very important aspects of large-scale collaborations, specialized diagnostic protocols and high-throughput analysis study designs are sample handling, shipping, and storing of samples which would all be easier with these simple protocols.

## Limitations

Self-sampling methods analysed in this study offer many advantages when compared to classic venepuncturing such as non-invasiveness, simplicity of sampling procedure, and accessibility. However, issues such as variety in sampled volume and Hct, haemolysis and relatively small sample volume may interfere with the analysis and influence the results. Another limitation of the study is relatively small sample size. Sample size of this study was determined by participant and material availability, therefore there was no prior calculation of power analysis. One more limitation of the study is the comparison of different starting samples—capillary whole blood (DBS and Neotreyx Mitra), dried plasma spots (Noviplex cards) and plasma from venepuncturing. However, studies have shown high uniformity of observed glycan structures in said samples [15–17].

## Supplementary Information

**Additional file 1:** **Table S1.** Abbreviations, major structures and descriptions of all glycans complementary to every plasma glycan peak. **Table S2.** Comparability of self-sampling methods with standard plasma method. **Figure S1.** Representative chromatogram with 39 separated glycan peaks acquired from plasma sample. **Figure S2.** Representative chromatogram with 39 separated glycan peaks acquired from Noviplex card (plasma separation disk). **Figure S3.** Representative chromatogram with 39 separated glycan peaks acquired from Mitra sticks (Neoterxy). **Figure S4.** Representative chromatogram with 39 separated glycan peaks acquired from DBS.

## Data Availability

The data used and analysed during the current study are available from the corresponding author on reasonable request.

## Abbreviations

2-AB: 2-Aminobenzamide
ACN: Acetonitrile
CV: Coefficient of variation
DBS: Dried blood spots
EDTA: Ethylenediaminetetraacetic acid
FLR: Fluorescence
GlcNAc: N-acetylglucosamine
GU: Glucose unit
HILIC: Hydrophilic interaction liquid chromatography
LC–MS: Liquid chromatography-mass spectrometry
PBS: Phosphate buffered saline
GP: Plasma glycan peak
RT: Retention time
SD: Standard deviation
SDS: Sodium dodecyl sulfate
SPE: Solid-phase extraction
UPLC: Ultra-performance liquid chromatography
