# Supplementary material for: Comparison of self-sampling blood collection for N-glycosylation analysis
Source: BMC Res Notes. 2022 Feb 16;15:61. doi: 10.1186/s13104-022-05958-9 (PMC8849020; doi:10.1186/s13104-022-05958-9)
Supplement: Supplementary file 1 — Additional file 1: Table S1. Abbreviations, major structures and descriptions of all glycans complementary to every plasma glycan peak. Table S2. Comparability of self-sampling methods with standard plasma method. Figure S1. Representative chromatogram with 39 separated glycan peaks acquired from plasma sample. Figure S2. Representative chromatogram with 39 separated glycan peaks acquired from Noviplex card (plasma separation disk). Figure S3. Representative chromatogram with 39 separated glycan peaks acquired from Mitra sticks (Neoterxy). Figure S4. Representative chromatogram with 39 separated glycan peaks acquired from DBS. [file 13104_2022_5958_MOESM1_ESM.docx]

**Additional Files**

**Comparison of self-sampling blood collection for N-glycosylation analysis**

Ana Cvetko^1^, Marko Tijardović^1^, Iva Bilandžija-Kuš^2^ and Olga Gornik^1^

^1^Faculty of Pharmacy and Biochemistry, University of Zagreb, Zagreb 10000, Croatia

^2^University Hospital Centre Zagreb, Zagreb 10000, Croatia

Corresponding author:

Olga Gornik

[ogornik@pharma.unizg.hr](mailto:ogornik@pharma.unizg.hr)

Faculty of Pharmacy and Biochemistry, University of Zagreb

A. Kovačića 1, 10 000 Zagreb, Croatia

*Deglycosylation, labelling and clean-up of plasma N-glycans*

Sample preparation for HILIC-UPLC-FLR analysis of labelled plasma N-glycans was done as described previously (27). This protocol was done in the same way for plasma samples, plasma disks obtained from Noviplex cards, DBS and blood extracted from Neoteryx Mitra sticks. Noviplex plasma disks and DBS remained in the wells until the SPE clean-up step. In short, 10 μL of samples were firstly denatured by 20 μl of 2% (w/v) SDS (Invitrogen, USA) and ten-minute 65 °C incubation, after which denatured samples were mixed with 10 μl of 4% (v/v) Igepal-CA630 (Sigma Aldrich, USA) and shaken for 15 minutes on a plate shaker (GFL, Germany). To cleave the N-glycans from the proteins, 1.2 U of enzyme PNGase F (Promega, USA) was added to the wells and incubated overnight at 37 °C. The following day, N-glycans were fluorescently labelled (25 μL/sample) with in-house prepared 2-aminobenzamide dye mixture (2-AB, 19.2 mg/mL; Sigma Aldrich, USA). Two-hour incubation at 65 °C followed to enhance the labelling step. Volume of 700 μL of 100% cold acetonitrile was then added to each sample and transferred to a preconditioned 0.2 μm GHP filter plate (Pall Corporation, USA). Sample clean-up based on HILIC-SPE was done by subsequently washing the samples 5× with 96% ACN using a vacuum manifold (Millipore Corporation, USA). Lastly, N-glycan elution was done 2x with 90 μL of ultra-pure water and stored at − 20 °C until UPLC analysis.

*HILIC-UPLC-FLR plasma N-glycan analysis*

Labelled N-glycans separation was done by hydrophilic interaction liquid chromatography (HILIC) on Acquity UPLC H-Class instrument (Waters, USA) as explained previously (27). Empower 3 software, build 3471 (Waters, Milford, USA) was used for instrument control. Separation of N-glycans was done on Waters BEH Glycan chromatography column, using 100 mM ammonium formate, pH 4.4, as solvent A and LC-MS grade acetonitrile as solvent B. Separation method characteristics and system calibration were described in detail previously (27). All samples were integrated manually and separated into 39 peaks (GP1–GP39). As a result of variation in laboratory conditions, signal intensities vary between samples. Therefore, each individual integrated peak area was normalized to total integrated area of the chromatogram (further referred to as (relative) abundancy), to allow for comparison of the samples.

*Statistical analysis*

For the comparability testing, relative deviance from plasma method was calculated using the following formula for each of 10 subjects:

$$R{elative deviance}_{GPn} = \frac{{GPn}_{tested method}-{GPn}_{plasma}}{{GPn}_{plasma}}$$

where GPn is the relative abundance of individual glycan peak (GP1-GP39). The procedure was repeated for results obtained from DBS, Neoteryx Mitra sticks and Noviplex plasma disks. For each method and GP, median of relative deviance between subjects was calculated and used in the following step. To represent the average relative deviance of each method, mean of each GP's relative deviance, as an absolute value, was calculated and reported. For the repeatability testing we have calculated CV % for each of 39 glycan peaks from hexaplicates for DBS, Neoteryx Mitra sticks and Noviplex plasma disks, and repeated it for all 5 participants. We then calculated median values of these CV % between participants, for each of the 39 glycan peaks for DBS, Neoteryx Mitra sticks and Noviplex plasma disks.

**Supplementary Table 1** Abbreviations, major structures and descriptions of all glycans complementary to every plasma glycan peak.*

| ***Sample*** | ***Glycan peak*** | ***Glycan structures (major)*** | ***Glycan structure description*** | ***Glycan peak abundance formula*** |
| --- | --- | --- | --- | --- |
| plasma | GP1 |  | core fucosylated, biantennary | GP1 / GP * 100 |
| plasma | GP2 |  | core fucosylated, biantennary with bisecting GlcNAc;  oligomannose | GP2 / GP * 100 |
| plasma | GP3 |  | monogalactosylated, biantennary with bisecting GlcNAc | GP3 / GP * 100 |
| plasma | GP4 |  | core fucosylated and monogalactosylated, biantennary | GP4 / GP * 100 |
| plasma | GP5 |  | core fucosylated and monogalactosylated, biantennary | GP5 / GP * 100 |
| plasma | GP6 |  | core fucosylated and monogalactosylated, biantennary with bisecting GlcNAc | GP6 / GP * 100 |
| plasma | GP7 |  | oligomannose;  core fucosylated and monogalactosylated, biantennary with bisecting GlcNAc | GP7 / GP * 100 |
| plasma | GP8 |  | digalactosylated, biantennary | GP8 / GP * 100 |
| plasma | GP9 |  | digalactosylated, biantennary with bisecting GlcNAc | GP9 / GP * 100 |
| plasma | GP10 |  | core fucosylated, digalactosylated, biantennary | GP10 / GP * 100 |
| plasma | GP11 |  | core fucosylated, digalactosylated, biantennary with bisecting GlcNAc | GP11 / GP * 100 |
| plasma | GP12 |  | oligomannose;  digalactosylated, monosialylated, biantennary;  tetramannosylated, monogalactosylated, monosialylated, monoantennary;  monogalactosylated, monosialylated, biantennary with bisecting GlcNAc | GP12 / GP * 100 |
| plasma | GP13 |  | core fucosylated, monogalactosylated and monosialylated biantennary;  core fucosylated, monogalactosylated and monosialylated biantennary with bisecting GlcNAc | GP13 / GP * 100 |
| plasma | GP14 |  | digalactosylated and monosialylated biantennary | GP14 / GP * 100 |
| plasma | GP15 |  | digalactosylated and monosialylated biantennary with bisecting GlcNAc | GP15 / GP * 100 |
| plasma | GP16 |  | core fucosylated, digalactosylated and monosialylated biantennary | GP16 / GP * 100 |
| plasma | GP17 |  | core fucosylated, digalactosylated and monosialylated biantennary with bisecting GlcNAc | GP17/ GP * 100 |
| plasma | GP18 |  | digalactosylated and disialylated biantennary;  core fucosylated, digalactosylated and disialylated biantennary | GP18 / GP * 100 |
| plasma | GP19 |  | oligomannose | GP19 / GP * 100 |
| plasma | GP20 |  | digalactosylated and disialylated biantennary | GP20 / GP * 100 |
| plasma | GP21 |  | digalactosylated and disialylated biantennary;  trigalactosylated and monosialylated triantennary;  core fucosylated, digalactosylated and disialylated biantennary;  digalactosylated and disialylated biantennary with bisecting GlcNAc;  antennary fucosylated, trigalactosylated and monosialylated triantennary | GP21 / GP * 100 |
| plasma | GP22 |  | core fucosylated, digalactosylated and disialylated biantennary | GP22 / GP * 100 |
| plasma | GP23 |  | core fucosylated, digalactosylated and disialylated biantennary with bisecting GlcNAc | GP23 / GP * 100 |
| plasma | GP24 |  | trigalactosylated and disialylated triantennary;  antennary fucosylated, trigalactosylated and monosialylated triantennary | GP24 / GP * 100 |
| plasma | GP25 |  | trigalactosylated and disialylated triantennary;  core fucosylated, antennary fucosylated, digalactosylated and disialylated biantennary;  antennary fucosylated, trigalactosylated and disialylated triantennary | GP25 / GP * 100 |
| plasma | GP26 |  | trigalactosylated and disialylated triantennary;  core fucosylated, trigalactosylated and disialylated triantennary | GP26 / GP * 100 |
| plasma | GP27 |  | antennary fucosylated, trigalactosylated and disialylated triantennary;  trigalactosylated and trisialylated triantennary | GP27 / GP * 100 |
| plasma | GP28 |  | trigalactosylated and trisialylated triantennary;  antennary fucosylated, trigalactosylated and disialylated triantennary | GP28 / GP * 100 |
| plasma | GP29 |  | trigalactosylated and trisialylated triantennary;  antennary fucosylated, trigalactosylated and disialylated triantennary | GP29 / GP * 100 |
| plasma | GP30 |  | trigalactosylated and trisialylated triantennary;  antennary fucosylated, trigalactosylated and trisialylated triantennary | GP30 / GP * 100 |
| plasma | GP31 |  | core fucosylated, trigalactosylated and trisialylated triantennary;  trigalactosylated and trisialylated triantennary | GP31 / GP * 100 |
| plasma | GP32 |  | trigalactosylated and trisialylated triantennary | GP32 / GP * 100 |
| plasma | GP33 |  | antennary fucosylated, trigalactosylated and trisialylated triantennary | GP33 / GP * 100 |
| plasma | GP34 |  | core fucosylated, trigalactosylated and trisialylated triantennary;  tetragalactosylated and trisialylated tetraantennary | GP34 / GP * 100 |
| plasma | GP35 |  | core fucosylated, antennary fucosylated, trigalactosylated and trisialylated triantennary;  antennary fucosylated, tetragalactosylated and trisialylated tetraantennary | GP35 / GP * 100 |
| plasma | GP36 |  | tetragalactosylated and trisialylated tetraantennary;  antennary fucosylated, tetragalactosylated and trisialylated tetraantennary | GP36 / GP * 100 |
| plasma | GP37 |  | tetragalactosylated and tetrasialylated tetraantennary;  antennary fucosylated, tetragalactosylated and trisialylated tetraantennary | GP37 / GP * 100 |
| plasma | GP38 |  | tetragalactosylated and tetrasialylated tetraantennary;  antennary fucosylated, tetragalactosylated and tetrasialylated tetraantennary;  antennary fucosylated, tetragalactosylated and trisialylated tetraantennary | GP38 / GP * 100 |
| plasma | GP39 |  | antennary fucosylated, tetragalactosylated and tetrasialylated tetraantennary;  antennary difucosylated, tetragalactosylated and tetrasialylated tetraantennary; | GP39 / GP * 100 |

*structure abbreviations – all N-glycans have two core *N*-acetylglucosamines (GlcNAcs); F at the start of the abbreviation indicates a core-fucose α1,6-linked to the inner GlcNAc; Mx, number (x) of mannose on core GlcNAcs; Ax, number of antenna (GlcNAc) on trimannosyl core; A2, biantennary with both GlcNAcs as β1,2-linked; A3, triantennary with a GlcNAc linked β1,2 to both mannose and the third GlcNAc linked β1,4 to the α1,3 linked mannose; A4, GlcNAcs linked as A3 with additional GlcNAc β1,6 linked to α1,6 mannose; B, bisecting GlcNAc linked β1,4 to β1,3 mannose; G(x), number (x) of β1,4 linked galactose on antenna; F(x), number (x) of fucose linked α1,3 to antenna GlcNAc; S(x), number (x) of sialic acids linked to galactose


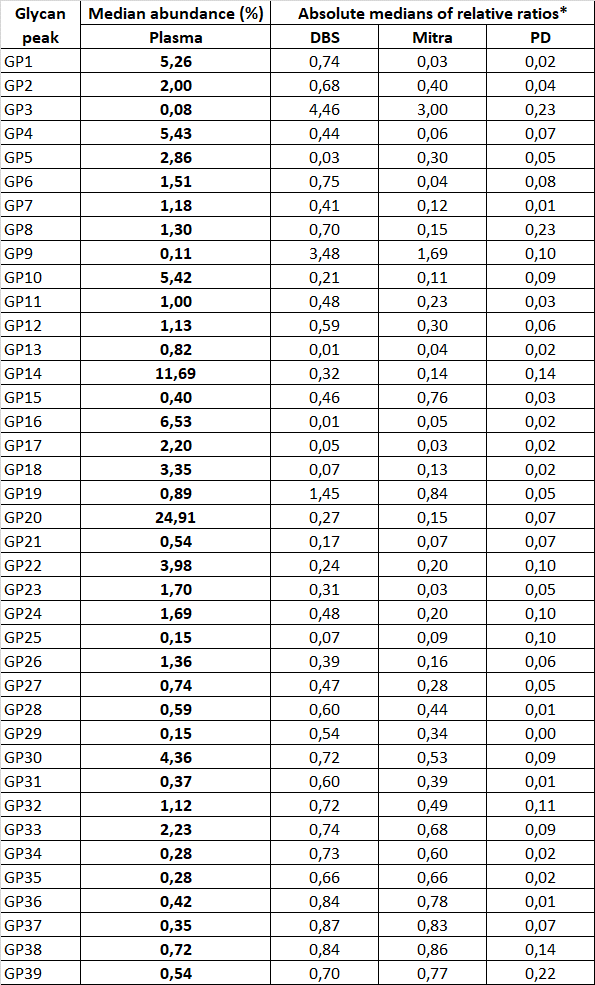
**Supplementary Table 2** Comparability of self-sampling methods with standard plasma method.

*Relative ratio: (self-sampling method value / standard plasma method value)


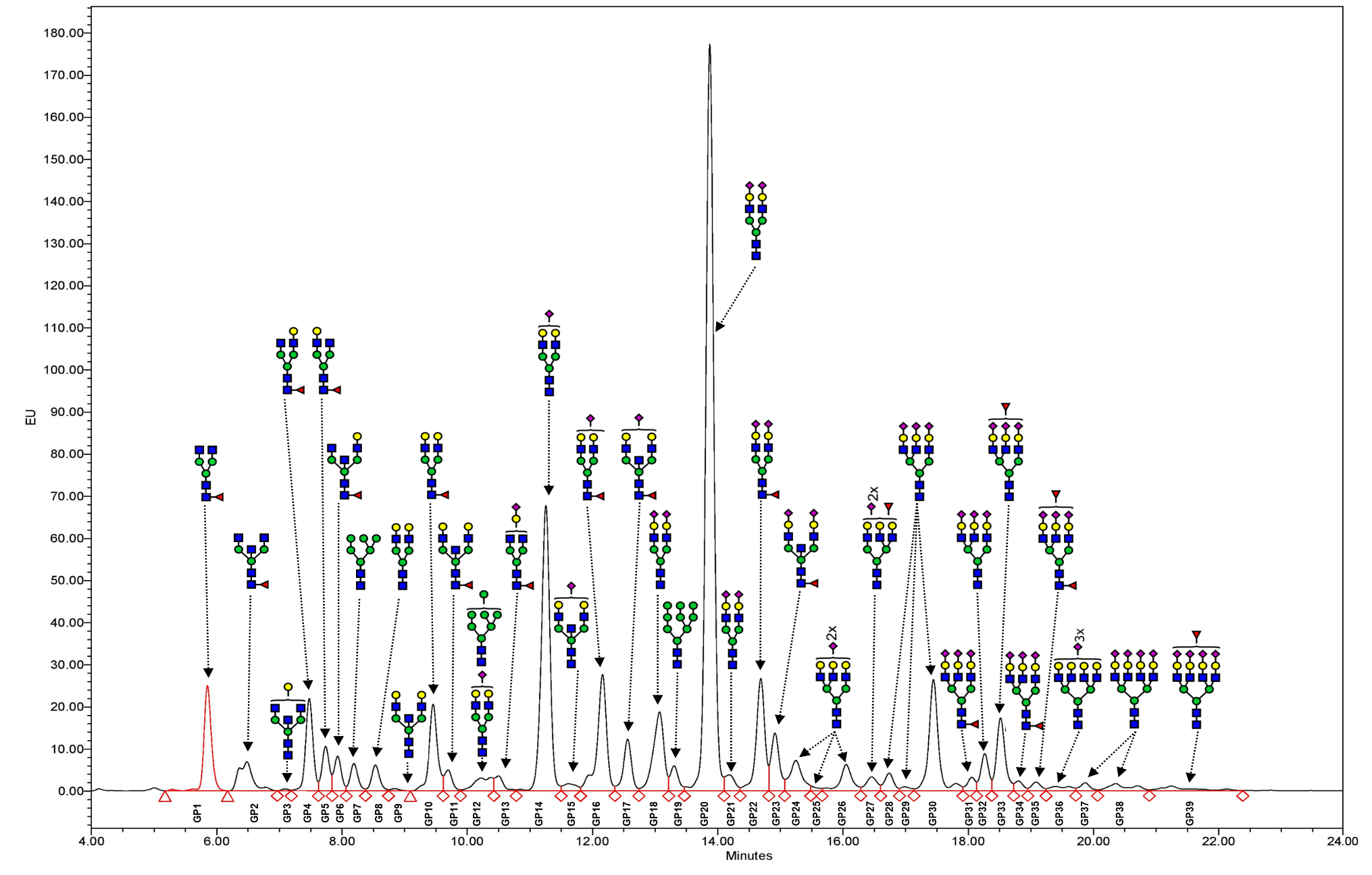


**Supplementary Figure 1** Representative chromatogram with 39 separated glycan peaks acquired from plasma sample.


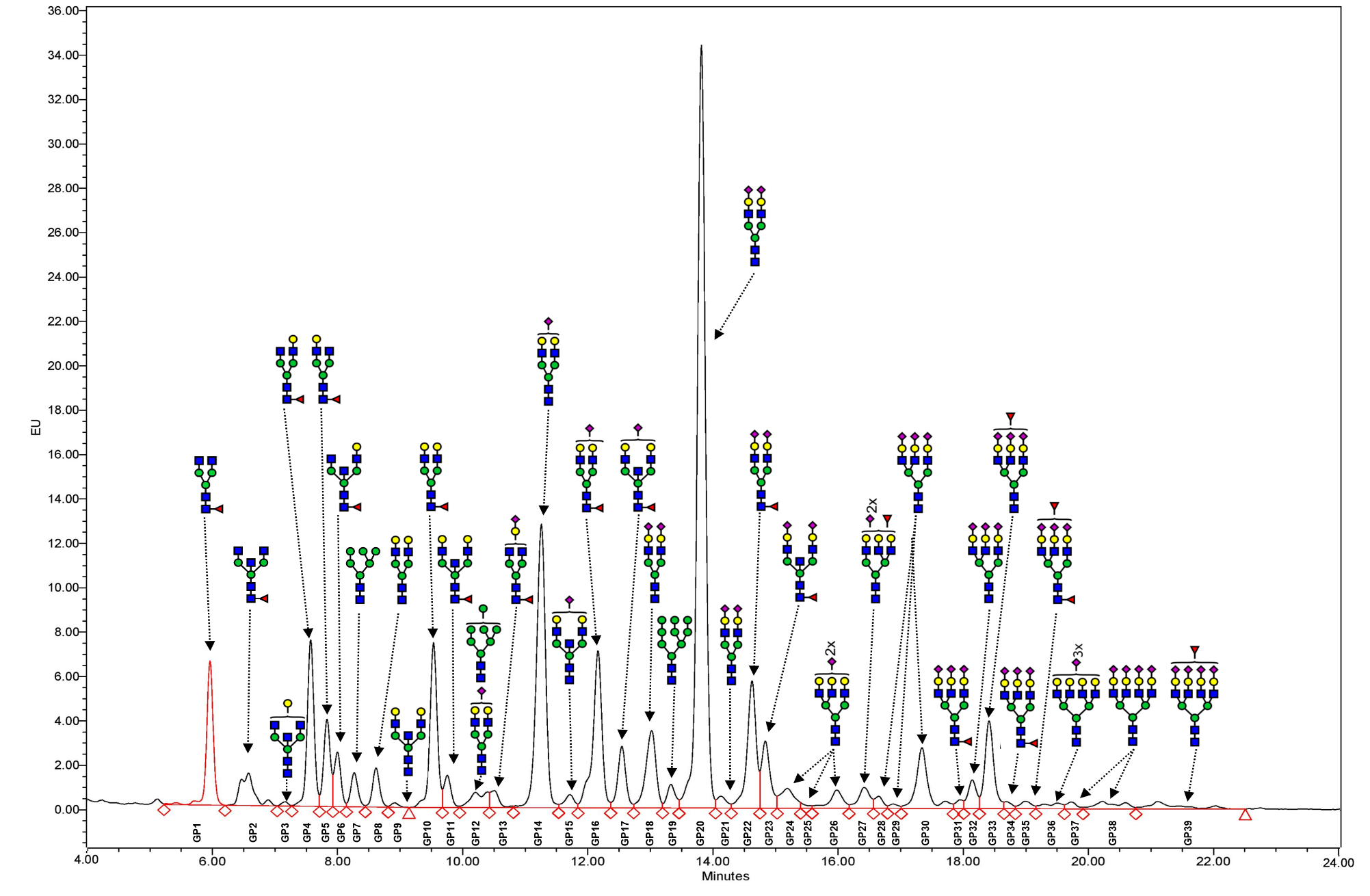


**Supplementary Figure 2** Representative chromatogram with 39 separated glycan peaks acquired from Noviplex card (plasma separation disk).


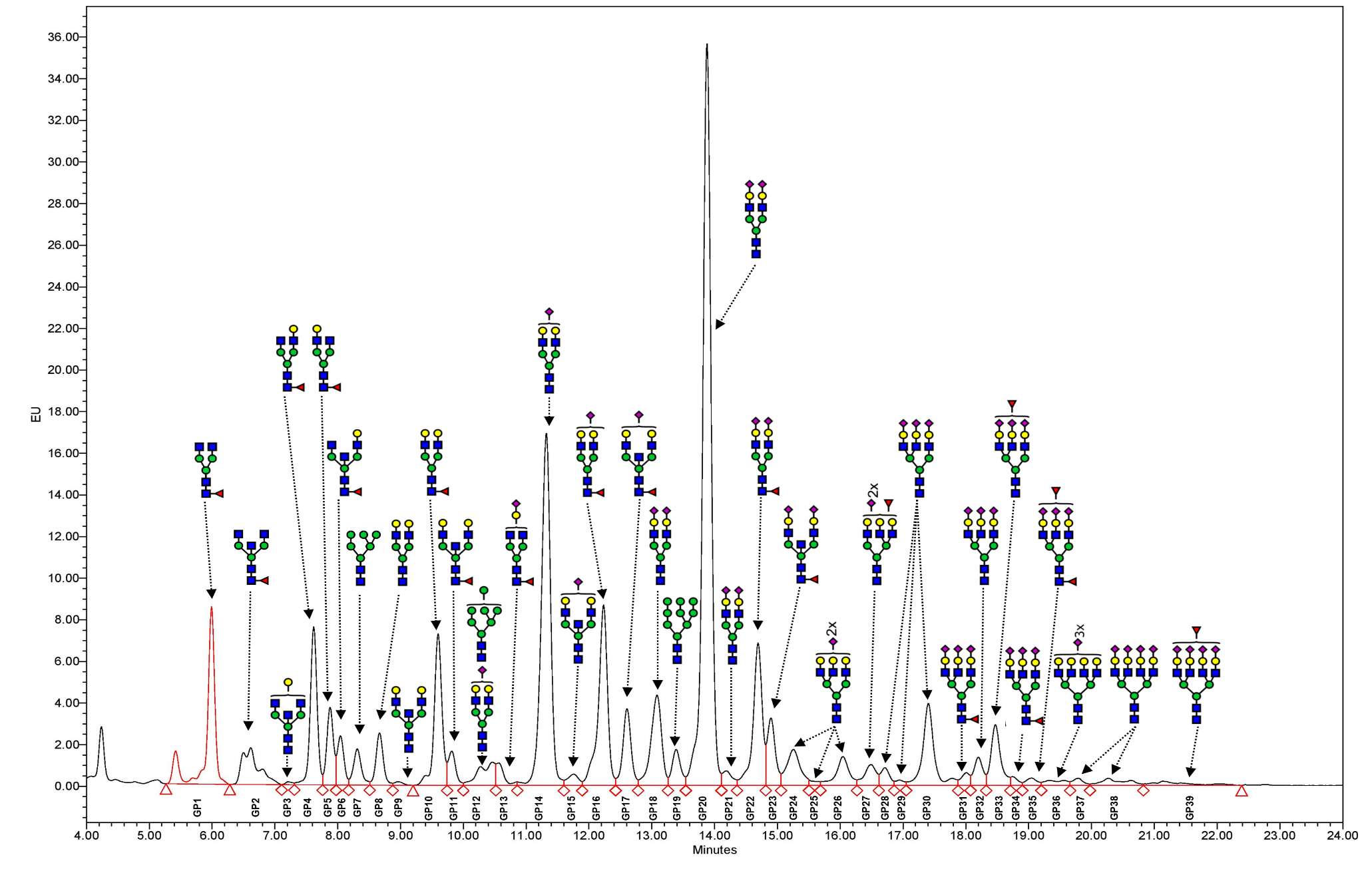


**Supplementary Figure 3** Representative chromatogram with 39 separated glycan peaks acquired from Mitra sticks (Neoterxy).


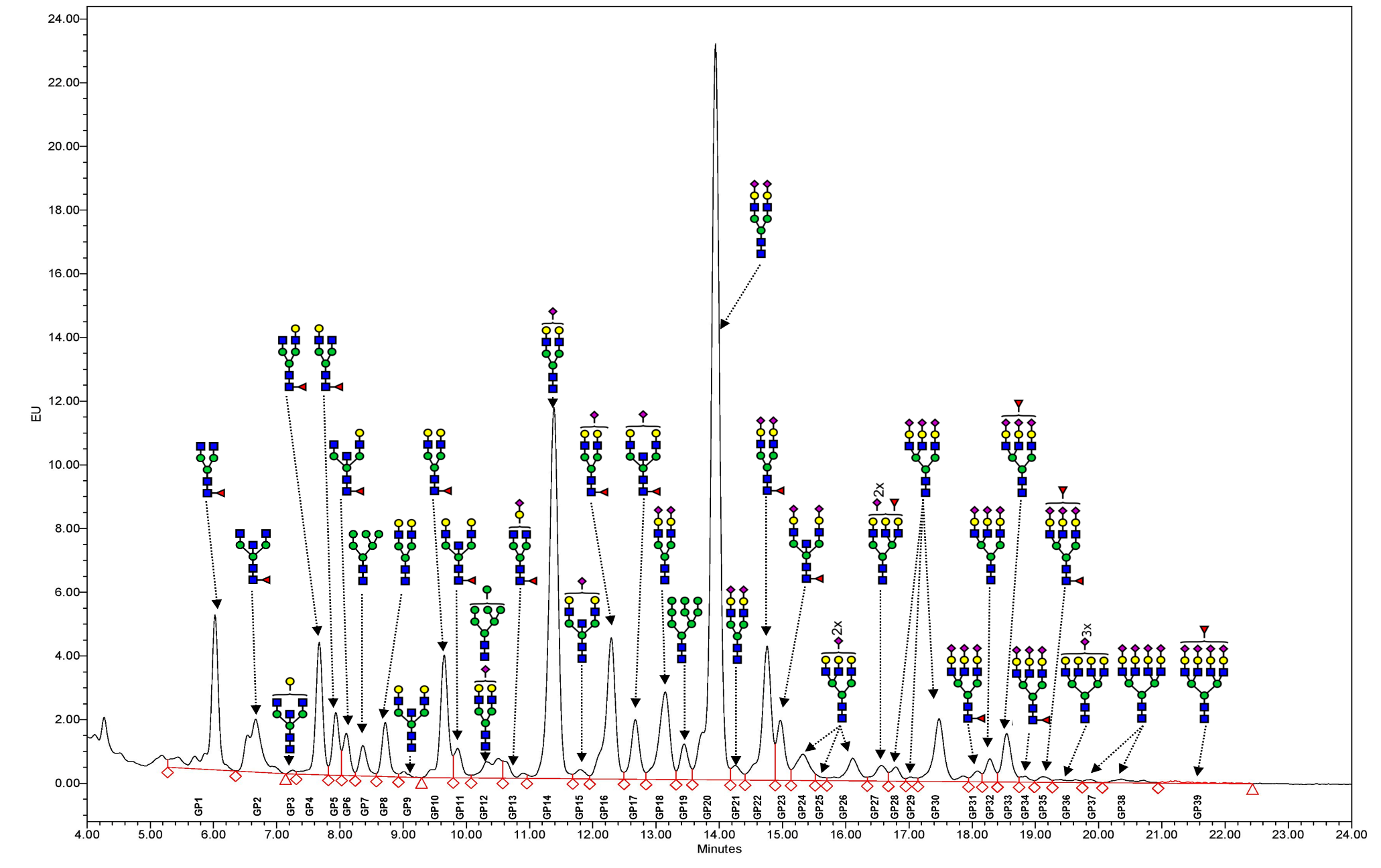


**Supplementary Figure 4** Representative chromatogram with 39 separated glycan peaks acquired from DBS.
